# Recent Advancements in Electrospun Chitin and Chitosan Nanofibers for Bone Tissue Engineering Applications

**DOI:** 10.3390/jfb14050288

**Published:** 2023-05-22

**Authors:** S Shree Ganesh, Ramprasad Anushikaa, Venkadesan Sri Swetha Victoria, Krishnaraj Lavanya, Abinaya Shanmugavadivu, Nagarajan Selvamurugan

**Affiliations:** Department of Biotechnology, School of Bioengineering, College of Engineering and Technology, SRM Institute of Science and Technology, Kattankulathur 603203, India

**Keywords:** biomaterials, biocomposites, bone tissue engineering, electrospun nanofibers, critical bone defects

## Abstract

Treatment of large segmental bone loss caused by fractures, osteomyelitis, and non-union results in expenses of around USD 300,000 per case. Moreover, the worst-case scenario results in amputation in 10% to 14.5% of cases. Biomaterials, cells, and regulatory elements are employed in bone tissue engineering (BTE) to create biosynthetic bone grafts with effective functionalization that can aid in the restoration of such fractured bones, preventing amputation and alleviating expenses. Chitin (CT) and chitosan (CS) are two of the most prevalent natural biopolymers utilized in the fields of biomaterials and BTE. To offer the structural and biochemical cues for augmenting bone formation, CT and CS can be employed alone or in combination with other biomaterials in the form of nanofibers (NFs). When compared with several fabrication methods available to produce scaffolds, electrospinning is regarded as superior since it enables the development of nanostructured scaffolds utilizing biopolymers. Electrospun nanofibers (ENFs) offer unique characteristics, including morphological resemblance to the extracellular matrix, high surface-area-to-volume ratio, permeability, porosity, and stability. This review elaborates on the recent strategies employed utilizing CT and CS ENFs and their biocomposites in BTE. We also summarize their implementation in supporting and delivering an osteogenic response to treat critical bone defects and their perspectives on rejuvenation. The CT- and CS-based ENF composite biomaterials show promise as potential constructions for bone tissue creation.

## 1. Introduction

**Figure 1:**
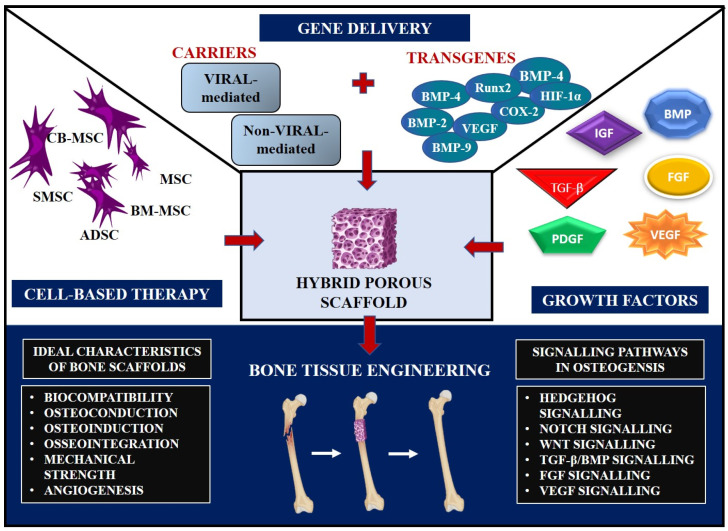
Schematic diagram of bone tissue regeneration through scaffold-based tissue engineering approaches. The tissue engineering triad can be concluded as utilizing a combination of scaffolds, cells, and growth factors.

Large bone deformities caused by extensive trauma, congenital musculoskeletal defects, infections, and cancer are often accompanied by severe complications. It is estimated that millions of people worldwide experience bone-anomaly-related fractures every year. The most ubiquitous musculoskeletal defects are fractures, which predominantly affect geriatric people around the age of 65 or over [1,2]. A critical-sized bone defect is defined as a minimal osseous defect with a length of more than two to five times the diameter of the injured bone that cannot heal naturally or exhibits less than 10% bone regeneration. These defects do not heal on their own without surgical intervention due to significant bone loss at the site of injury, which adversely affects vascularization and bone differentiation [3]. A gold-standard therapy for such defects is to employ autologous bone grafts, which are most commonly procured from the iliac crest or by allografts; however, upon usage, this technique exhibits several shortcomings, such as problems with healing and lack of capacity to meet demand, along with reports of infection, hematomas, re-operative proceedings, sustained bone graft discomfort, wound drainage, sensory loss, and keloids [3,4]. To address these issues, an effective method for bone substitution is required, and the field of bone tissue engineering (BTE) opens a new possibility to alleviate such hurdles by uniting cells, biological factors, and scaffolds for the regeneration of critical-sized bone defects (Figure 1) [5]. This approach averts complications and provides beneficial traits such as modifiability, biocompatibility, and reduction in occurrences of infection [6]. One of the essential aspects of biomaterial selection is biocompatibility, which is described as “the capacity of a biomaterial to execute its function without provoking toxic or harmful effects on biological systems while generating a suitable response from the host in a specific case”. Therefore, biocompatibility testing is an important prerequisite for regulatory agencies in creating and approving orthopedic materials for clinical use [7,8]. Scaffolds are the three-dimensional (3D) networks replicating extracellular matrix (ECM) and facilitate cell adhesion, proliferation, and development in vitro while preserving the basic structure of regenerated tissue in vivo. Additionally, if the bone is repaired correctly, the bone scaffold will be less likely to fail, saving the patient from needing additional surgery. Bone scaffolds can enable patients to continue engaging in their preferred activities, which can save patient costs, shorten the duration of their hospitalizations, and improve their quality of life [9]. To successfully fabricate a scaffold, three key factors play a pivotal role; (1) the biomaterial to be employed, (2) the type of fabrication technique for production, and (3) the structure to be replicated. Based on their composition, biomaterials can be classified as bioceramics, polymers, metals, or composites [10]. Ceramics offer numerous advantages, including similarity to the mineralogical composition of native bone and osteoconductive properties; however, their application is limited due to their brittle nature and difficulty in processing [11]. When it comes to polymer-based scaffolds, they can be procured from both synthetic and natural sources. Polysaccharides such as alginate (Alg), chitin (CT)/chitosan (CS), hyaluronic acid (HA) and derivatives, as well as proteins such as collagen (Col), fibrin gels, and silk, are examples of naturally occurring polymers. Synthetic polymers have gained considerable attention due to their processability, mechanical abilities, and lack of immunogenicity [12]. However, compared with synthetic polymers, natural polymers exhibit enhanced biocompatibility and bioactivity [13]. Two of the most prevalent natural polymers available, CT and CS, have garnered a lot of interest as bone substitutes owing to their non-toxic, antibacterial, biocompatible, and degradable attributes and can be acquired from the shells of crustaceans [14].

It is essential to precisely choose a biomaterial of interest and a suitable fabrication technique according to the specific tissue application [15]. Scaffolds come in various forms, including decellularized matrix, bio-printed, 3D-printed, freeze-dried and nanofiber (NF) scaffolds [10]. Among these, the production and application of NFs in the biomedical field are in their exponential phase [16]. NF scaffolds hold distinct physical and chemical attributes that contribute to their exceptionally high surface-area-to-volume ratio, minuscule pore size, and high porosity; moreover, NF scaffolds play a critical role in BTE and have proven to effectively mimic the structure of the natural bone matrix, encouraging cell proliferation and stimulating osteogenesis for bone regeneration [14]. ENFs are also capable of acting as vascular grafts, aiding in neovascularization. This attribute reportedly supports the osteogenesis process by mobilizing progenitor cells [17,18]. Various techniques can be used to create CT and CS NFs [19]. The technical difficulty added to the expenditure, minimal yield, and lack of control over the size of the resulting fibers place a limit on these fabrication methods for the production of NFs. Hence, electrospinning has been proposed as a practical, adaptable, and rapid fabrication method as an effective alternative for creating continuous polymer fibers with sizes extending from nanometers to microns [20]. The electrospinning is performed using a simple protocol where a polymer solution is loaded into a syringe and subjected to an applied voltage; when the supplied electric potential overcomes the surface tension, the droplet extends to form fibers that get collected on the collector as non-woven fiber as it dries. The produced NFs exhibit high surface-area-to-volume ratio, high porosity, and intrinsic mechanical properties and mimic the ECM in vivo [11,21]. In this review, we predominantly discuss electrospun CT/CS NFs, their derivatives, applications, and therapeutic prospects in the field of BTE. By breaking the conventional ideas about the execution of multifunctional electrospinning and the approach of integrating surface properties and inner structural features, the current review opens a new path to exploring material processing techniques and producing novel functional ENF constructs for BTE.

## 2. Chemical Structure of Chitin and Chitosan

### 2.1. Biomaterials

Biomaterials or biomedical materials play a significant role in scaffold fabrication and BTE applications. Scaffolds are used in BTE as they mimic the properties of natural bone ECM and enhance other properties by providing the 3D environment required for bone repairs such as adhesion, proliferation capacity, and differentiation [22]. A biomaterial is expected to be non-cytotoxic, biocompatible, biodegradable, and easily printable. In addition, BTE requires biomaterials to have certain qualities that aid osteoconduction, osteoinduction, and osteointegration [23]. Polymers are flexible biomaterials that can be molded into any shape as required. They have a desirable load-bearing capability, aid in skeletal attachment, and support osteogenesis, making them a promising biomaterial for BTE applications [22,24].

Polymers can be further classified as natural or synthetic [25]. Synthetic polymers have excellent crystallinity and modifiable mechanical and physical properties. The polymers that are listed under synthetic polymers are polylactic acid (PLA), polyglycolic acid (PGA), polylactic-co-glycolic acid (PLGA), polycaprolactone (PCL), polyvinyl alcohol (PVA), propylene fumarate (PPF), etc. These polymers are able to produce bone constructs with customizable shapes, porosities, and degradation rates. Some disadvantages are the adverse effects on tissues due to acidic degradation and a shortage of cell adhesion ability. In contrast to synthetic polymers, natural polymers possess biocompatibility, similarity to ECM, biodegradability, and desirable biological and mechanical properties, making them efficient polymers to be used in scaffolds. Some naturally derived polymers are gelatin (Gel), silk fibroin (SF), Col, CT, CS, Alg, and HA [17]. Among these natural polymers, CT and CS have drawn keen attention as bone substitute biomaterials due to their non-toxicity, antibacterial activity, biocompatibility, and degradability [14,23].

### 2.2. Chitin and Its Structure

CT is a natural cationic polysaccharide that is a glycosaminoglycan; glycosaminoglycans are a key element found in the ECM of bone. CT is typically found in residual ocean biomass, including crab, shrimp, or lobster dumped in coastal areas, and also found in insects, fungus, mushroom envelopes, yeast, and green algae [14,26]. CT is widely known for its use in the field of bioengineering since it is biodegradable, biocompatible, non-toxic, renewable, and has antibacterial properties. It can be fabricated into fibers, films, and aero/hydrogels [14,26,27,28]. CT comprises 2-acetamido-2-deoxy-β-D-glucose through a β (1 → 4) linkage. It consists of 6–7% nitrogen. X-ray diffraction studies revealed that CT is a polymorphic material with three distinct crystalline modifications, namely α-, β-, and γ-CT [28]. α-CT is found predominantly in arthropods, fungi, Entamoeba cysts, chitinous cuticles, peritrophic matrices of insects, and crab and shrimp shells. It is arranged in an anti-parallel manner. β-CT is present in the peritrophic matrices of insects, molluscs, and the pen of the Loligo squid. It is arranged in a parallel manner. γ-CT consists of two parallel strands and one anti-parallel strand. It is found in cocoons and the stomachs of the Ptinus beetle and Loligo [29]. The arrangement of α-CT allows it to become tightly packed in the CT microfibrils, which consist of almost 20 CT chains stabilized by many hydrogen bonds within and between them, contributing to physiochemical properties such as mechanical strength and stability of the cuticle [30]. However, the arrangement of β- and γ-chains shows a reduced number of hydrogen bonds within and between them, resulting in reduced packing tightness. The increased number of hydrogen bonds with water makes them more flexible and softer structures. These forms can also be converted from one form to another, i.e., the β form can be converted to the α form and the γ form can be converted to the α form with lithium thiocyanate treatment [31].

### 2.3. Chitosan and Its Structure

**Figure 2:**
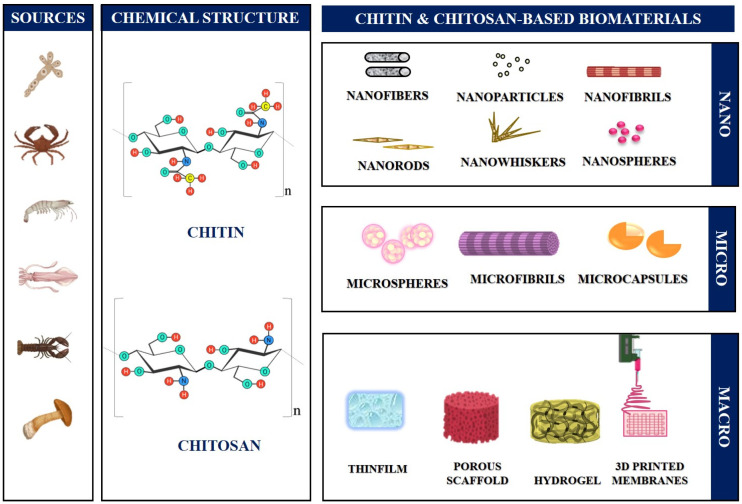
Schematic diagram illustrating the sources, chemical compositions, and various macro, micro, and nanoforms of CT/CS-based bone constructs used in BTE applications.

CS, a CT derivative, is produced via deacetylation of the natural polymer of CT. CS is biodegradable, biocompatible, antioxidant, non-toxic, and antibacterial. It is made up of glucosamine and *N*-acetyl glucosamine units joined by (1–4) glycosidic linkages [25,32]. CS is used in the field of biomedicine, agriculture, cosmetics, and food processing because of its capacity to develop into various forms such as films, NFs, nanoparticles, nanocapsules, microparticles, membranes, sponges, scaffolds, and hydrogels [33]. CS is derived by the deacetylation of α-CT with alkaline treatment at 100–160 °C in 40–50% aqueous alkali solution. CS consists of three crystal types similar to CT named as α, β and γ, of which the α type is more significant. CS contains three functional groups: amino/acetamido and primary and secondary hydroxyl groups, which are found in the CS polymer’s C-2, C-3, and C-6 positions. It consists of 2-acetamido-2-deoxy-β-d-glucopyranose and 2-amino-2-deoxy-β-d-glucopyranose. The properties of CS are governed by its molecular weight, degree of deacetylation, purity of the product, and the sequence of the amino and acetamido group present in it [23,34]. The sources, chemical makeups, and diverse CT/CS-based bone constructs employed in BTE applications are represented schematically in Figure 2.

## 3. Different Scaffold Fabrication Methods

Scaffolds act as a platform for cell adhesion, proliferation, migration, and differentiation. In BTE, there is a greater demand to fabricate scaffolds with porous structures since this can enhance bone regeneration by augmenting the surface area for cellular attachment and improving protein adsorption specific to the bone. Similarly, porosity also influences the reactivity of the scaffolds, ionic dissolution [35], and permeability, which enhances vascularization, nutrient exchange, oxygen flow, and waste elimination [36]. Biodegradation of the scaffolds also depends on their porous nature; degradation is faster when there is larger porosity. Scaffolds with pore sizes above 50 µm are categorized as macroporous, whereas those with pores under 50 µm are categorized as microporous. The macroporous structure allows cell penetration, enhancing tissue integration and in-growth, whereas the microporous structure helps maintain the scaffolds’ mechanical stability [37]. According to reports, the ideal pore size for enhanced osteogenic characteristics is 200 µm or greater [35,36,37]. The scaffold’s morphology, including porosity, can be attributed to fabrication technologies [6,9]. Freeze-drying (FD), phase separation, decellularized bone matrix, gas foaming, solvent casting particulate leaching, 3D printing, and electrospinning are some of the prevailing fabrication techniques for preparation of CT- and CS-based scaffolds and are discussed below.

### 3.1. Freeze-Drying

**Figure 3:**
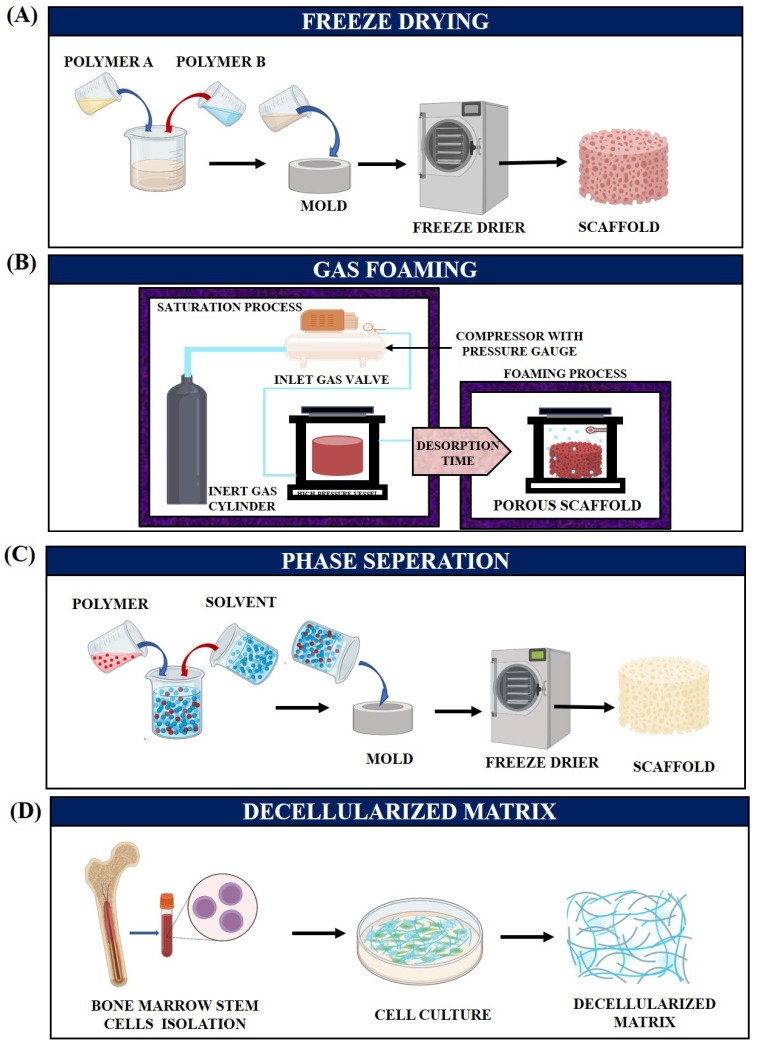

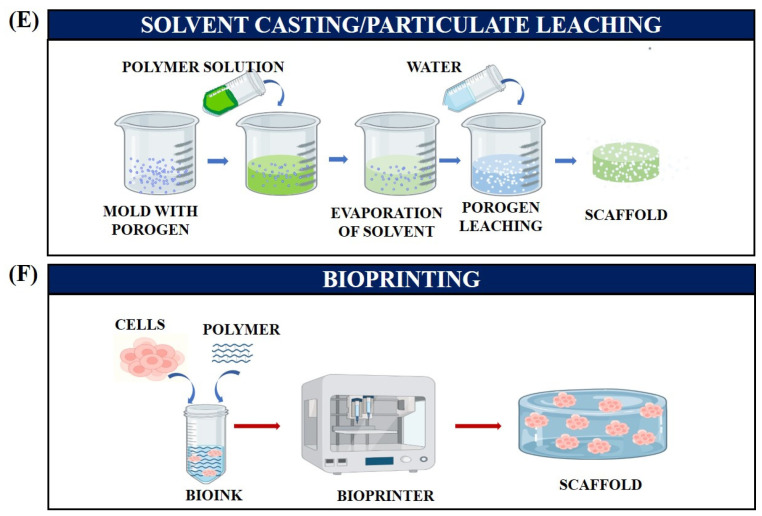
Diagrammatic representation of various types of scaffold preparation techniques. (**A**) Freeze-drying, (**B**) Gas foaming, (**C**) Phase separation, (**D**) Decellularized matrix, (**E**) Solvent casting followed by particulate leaching and (**F**) Bioprinting.

FD is a conventional technique that empowers fabricating 3D porous scaffolds with a porosity of over 90% and a pore diameter span of 20–400 µm [38]. It is a three-step method that involves preparing a solution to be freeze-dried, freezing the prepared solution at −20 °C to −80 °C, and lyophilizing it in a negative pressure environment (Figure 3A). Haghbin et al. created biodegradable PCL/CS/Gel porous scaffolds, which showed better mechanical properties, with maximum strength (25 MPa), a favorable modulus (3.86 MPa), and a faster deterioration rate than other scaffolds. Cell survival and adhesion were verified, demonstrating their potential application as a bone substitute. In order to replicate the bone ECM, FD methodology was exploited to develop a highly porous scaffold with a porosity level of more than 90% [39].

### 3.2. Gas Foaming

Gas foaming is a well-established technique employing supercritical fluid that functions as a plasticizer, limiting its glass transition and/or melting temperature to develop porosity within a 3D configuration (Figure 3B). Gas foaming is recurrently used as an effortless method to alchemize a 2D fiber membrane into a 3D porous structure, which is superior for applications that demand complete filling of the injured site [40,41]. Kim et al. reported that a 3D hierarchical multilayer scaffold modified by a gas-foaming technique provides an additional layer of functionality. Correspondingly, adding calcium to the 3D NF scaffolds aided cellular penetration and mineralization. The 2D electrospun mat was reconstructed into a 3D one via gas foaming. The 3D expansion was caused by gas entrapment in the inter-fiber junctions [42].

### 3.3. Phase Separation

There are several methods of phase separation, including diffusion-induced phase separation (DIPS) and thermally induced phase separation (TIPS), both of which enable the foams to be customized in terms of mechanical attributes and pore size for tissue engineering applications. A phase separation process employs the thermodynamic de-mixing of a homogeneous polymer–solvent (binary) or polymer–solvent–nonsolvent (ternary) solution to produce a polymer-rich (high polymer concentration) or a polymer-lean (low polymer concentration) material (Figure 3C). Furthermore, the solidification of the polymer-rich phase generates a solid matrix, whereas solvent removal induces the polymer-lean phase to yield porosity [43]. For instance, Singh et al. created scaffolds for BTE applications using nano bioglass encapsulated in CS/chondroitin sulphate complex by polyelectrolyte complexation/phase separation and resuspension of the separated complex into a Gel matrix. Phase separation was employed to augment the hydrogen, covalent, and ionic bonding of the constituents [44].

### 3.4. Decellularized Matrix

Decellularized ECM preparation is another fabrication technique that mimics a non-immune environment by using native 3D scaffolds and a multifarious bioactive component. These 3D scaffolds also overcome the quotidian encumbrances of conventional scaffolds, such as immunogenicity, simulating an in vivo microenvironment, and exhibiting mechanical or biochemical properties that closely resemble endemic organs and tissues [45]. In the process of decellularization, cells are amputated from the tissue by employing surfactants and enzymatic methods well as thermal shock, sonication, and hydrostatic pressure procedures while preserving the native ECM composition and architectural integrity (Figure 3D) [10]. Nyberg et al. effectuated 3D-printed PCL scaffolds employing a fused deposition modelling (FDM) process and functionalized them with multiple mineral additives such as tricalcium phosphate (TCP), HAp, Bio-Oss (BO), or decellularized bone matrix, wherein, PCL decellularized bone matrix exhibited superordinate competence for osteoinduction compared with synthetic materials and could also be an exceptional aid for bone recovery in vivo [46].

### 3.5. Solvent Casting/Particulate Leaching

The solvent casting/particulate leaching fabrication method is a standard technique that comprises an insoluble salt annexed to the polymer solution after it has been dissolved in the appropriate solvent (Figure 3E). A salt–polymer composite can be established by evaporating the solvent and washing the salt particles aside; this is a simple and straightforward process that does not demand the aid of extravagant and complex equipment. In addition, the technique allows for precise control of the final porosity (pore size, interconnectivity) and pore density by a proper selection of the polymers, porogens, and their respective amounts [47]. Huang et al. devised a porous scaffold with graphene platelets and salt (NaCl) that was exploited to create porous biomedical scaffolds by employing a solvent casting/particulate leaching fabrication technique. The prepared scaffolds showed much higher porosity (more than 85%; pore size: 250–400 µm) and more interconnected structures than those produced using other fabrication procedures [48].

### 3.6. 3D-Printing Techniques

Three-dimensional (3D) printing, also known as additive manufacturing (AM) and rapid prototyping, connects materials to form products from 3D model data. Numerous 3D printing manufacturing techniques have been evolved based on different working principles, including FDM, selective laser sintering (SLS) and stereolithography, inkjet 3D printing, adhesive droplet and powdered-bed-based AM, digital laser processing, and continuous liquid interface production [49,50]. Du et al. created a femoral-shaped porous scaffold bio-nanocomposite comprising of wollastonite (WS)/HAp/CS/PCL by utilizing 3D printing and FD technology for an orthopedic framework that enables an interim environment for bone development and fosters cell adhesion and differentiation [51].

Bioprinting is an advanced type of 3D printing technology involving cell-encumbered scaffolds in which bone constituents are conglomerated to form a 3D environment by employing bio-ink (Figure 3F) [52]. The microstructural, mechanical, and bioactive properties of CT-based composite scaffolds reinforced with akermanite have been evaluated. With the aid of spray-drying and subsequent heat treatment methods, the homogeneous and narrow particle size allocation of akermanite powders were attained. The scaffolds showed better mechanical strength and open, uniform, and interconnected pore morphology. Notably, good apatite-formation competence on the scaffold surfaces was discovered, which aided in demonstrating good in vitro bioactivity [53]. Correspondingly, Liu et al. fabricated CT whiskers/poly (L-lactide) composite scaffolds using a direct tri-solvent-ink writing 3D printer. CT whiskers were employed in this study owing to their outstanding mechanical properties, excellent cell affinity, and osteogenic attributes [54]. Table 1 summarizes some different scaffold fabrication techniques that are used in BTE applications.

## 4. Electrospinning Method

**Figure 4:**
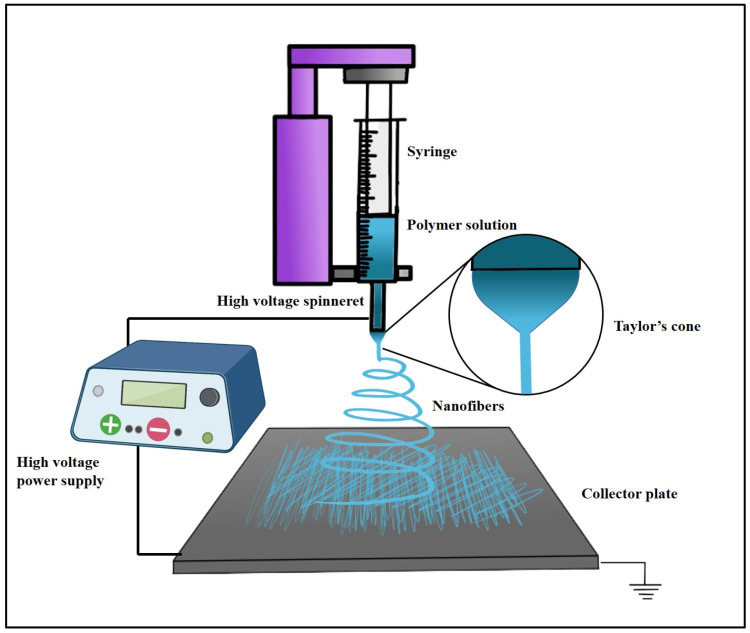
A schematic representation of the ES process technique.

In contrast to other fabrication techniques, electrospinning results in scaffolds with enhanced cellular adhesion, proliferation, and differentiation. These characteristics are likely due to the high specific surface-area-to-volume ratio afforded by a low-dimensional fibrous structure. Moreover, electrospinning is captivating, owing to its competence to manufacture NFs with structural resemblance to the native ECM, which may also enhance angiogenesis in different tissues [78]. Electrospinning is an accustomed fabrication technique that has been regarded as a process of developing microfibers to NFs from polymeric solutions at atmospheric pressure and room temperature by employing an elevated electric field (kV). The three cardinal components of an electrospinning device are the power supply (high voltage), the syringe (spinneret), and the collector (electrode) [79]. A schematic representation of the ES technique process is depicted in Figure 4.

The electrospinning fabrication process consists of several factors, such as the electrospinning parameters: which include (1) an applied electric field, (2) the needle-to-collector distance, and (3) the flow rate [80]; solution parameters: which encompass (1) polymer concentration—at low concentrations, charged jets lose intermolecular attraction due to increased surface tension; when the concentration is too high, an admixture of beads and fiber is formed, (2) viscosity—low-viscosity solutions cannot form continuous fibers, whereas high-viscosity solutions cannot generate enough electrical charge to attenuate the solution enough to form fibers; the notional spinning viscosities vary from 1 to 200 poise, yet 1 to 20 poises can create uniform NFs [81], and (3) solution conductivity—a drop surface with low conductivity does not form a Taylor cone; augmenting solution conductivity helps to initiate the process but exceeding the critical value can impede Taylor cone production and electrospinning processes [82].

The environmental parameters involve: (1) Relative humidity: elevated humidity promotes the fabrication of porous NFs, which facilitates cell adhesion and invasion due to evaporative cooling of the solvent. Humidity directly affects pore size and diameter. (2) Temperature: Higher temperatures produce NFs with reduced diameters, which can lead to early fiber drying before reaching the collector and drastically affect polymeric solution viscosity. Biological substances at higher temperatures can lose their functionality. At lower temperatures, solvent removal from the needle tip is slower than evaporation and clogs the needles during production [81,83]. Electrospun fibrous scaffolds can be rendered by harnessing multiple electrospinning tools that may be based on (i) needleless electrospinning and (ii) needle-based electrospinning.

### 4.1. Needleless Electrospinning

Needleless electrospinning refers to a process of fabricating NFs in which a polymeric solution is electrospun directly from a liquid surface without using needles. Needleless electrospinning systems have been developed to boost NF production rates and circumvent challenges such as needle clogging, low production, and limited manufacturing capability that are inherent to the traditional electrospinning process [79]. Multiple needleless electrospinning methods are listed below.

#### 4.1.1. Bubble Electrospinning

Bubble electrospinning works on the principle of injecting pressurized air or nitrogen (N2) into a polymeric solution, which leads to the formation of bubbles on the solution’s free surface; upon bursting, these bubbles extend out into many jets, at which point electrospinning is initiated. Unlike conventional electrospinning, instead of emanating from the tips of Taylor cones, the jets in bubble electrospinning extend out from the bubbles themselves [79]. The periodic wrinkled structure of bubble-electrospun nanofibers (ENFs) has diverse applications in adsorption, separation, screening, catalysis, fluid storage, and delivery. These investigations have far-reaching ramifications for various contexts, including radiation shielding, medical implants, cell supports, and materials that can be employed as instructive 3D environments for tissue regeneration [84].

#### 4.1.2. Wire Electrode Electrospinning

The process of wire electrospinning is categorized into three distinct phases: (i) the liquid is put onto the wire as it moves through a liquid–air interface, (ii) an annular layer of liquid breaks into droplets on the cylindrical wire, and (iii) the jets are formed from the droplets due to electrostatic forces [85]. Despite the fact that a spiral wire spinneret often necessitates more voltage than a needle electrospinning setup, the resulting fibers consistently have higher quality and lower diameters [79].

### 4.2. Needle-Based Electrospinning

#### 4.2.1. Monoaxial Electrospinning

Owing to its ease of fabrication, monoaxial electrospinning has numerous potential applications. Amorphously dispersed drug molecules are frequently found within the resulting fibers, and thus, monoaxial-electrospun fibers are well equipped to address the solubility challenges that plague many pharmaceutical constituents and create fast-dissolving drug-delivery systems. This principle follows the basic electrospinning principle [86]. Pangon et al. fabricated a bionanocomposite scaffold by utilizing CT whiskers CTWK to emphasize the mechanical properties of CS/PVA ENFs and to induce osteoblast cell development by mineralizing HAp. Because porous materials with explicit NF morphology can be effortlessly and effectively manufactured via electrospinning from a wide range of natural biopolymers, including proteins and polysaccharides, electrospinning was used for scaffold fabrication [87].

#### 4.2.2. Coaxial Electrospinning

Coaxial electrospinning is the process of fabricating ENFs in a coaxial arrangement to cover one fiber with another. The novel modification, rather than the conventional electrospinning setup, is based on the structure of a few capillary tubes positioned coaxially in the spinneret. There are two separate reservoirs of polymer solvent, one for the core and one for the shell layer [88]. Sruti et al. fabricated veratric-encapsulated CS nanoparticles embedded onto a PCL/PVP coaxial-electrospun system for continuous drug release for the induction osteoblast differentiation. In this investigation, coaxial electrospinning was employed as it provided a promising route for long-term drug delivery. The core–sheath configuration shields the capricious bioactive molecules or drugs from the oppressive environment and averts burst discharge [89]. Wang et al. employed coaxial electrospinning to create nanofibrous scaffolds to encourage bone defect regrowth by using magnesium-doped mesoporous bioactive glass (MBG) with a fusion protein of osteocalcin–osteopontin–biglycan (OOB), silk fibroin (SF), and nerve growth factor (NGF). They discovered that OOB@MBG/NGF@SF scaffolds could significantly enhance BMSC osteogenesis by promoting the Erk1/2-activated Runx2 and mTOR pathways and increasing the expression of osteogenic marker genes [90].

#### 4.2.3. Triaxial Electrospinning

The spinneret used in triaxial electrospinning has three concentric needles. The polymer solution employed is divided into three layers, i.e., inner, middle, and outer [91]. The deformation of the polymer solution occurs with the help of an electrostatic field in a Taylor cone fashion [92]. Triaxial electrospinning’s spinneret can be utilized to conduct one fluid uniaxial, two fluids coaxial, and three fluids coaxial electrospinning, producing monolithic, core-shell, and trilayer core-shell nanofibers [93]. Wang et al. effectively constructed a trilayer core-shell NF with a drug-free polymeric coating and an inner drug gradient distribution. Then they compared it with bilayer core-shell and monolithic medicated NFs. In vitro and in vivo gavage tests on rats revealed that trilayer NFs with elaborate structure designs provided a better sustained drug release profile than bilayer core-shell nanofibers regarding initial burst release, later tail-off release, and long-sustained release period [94].

#### 4.2.4. Side-by-Side Electrospinning

Side-by-side structures, which are more appealing for the fabrication of multifunctional nanoproducts than core-shell structures, are widely found in nature and have recently become a study hot topic for researchers. Side-by-side electrospinning involves a complicated combination of fluid dynamics, electrodynamics, and rheology, posing a considerable challenge for managing the simultaneous movement of two fluids in a side-by-side manner under an electrical field from the spinneret to the collector [92]. Peng et al. reported side-by-side electrospinning to create a homogeneous bio-based PLLA and Bombyx mori silk fibroin two-in-one fiber. These silk-based ENFs with β-sheet structures have a tensile strength of 16.5 ± 1.4 MPa, a modulus of 205 ± 20.6 MPa, and an elongation rate at a break of 53 ± 8%. It would be captivating to utilize such fibers to offer a novel platform for creating multiple-functional components and developing novel nanostructures, which could then be applied in a variety of areas such as biodegradation studies, cell culture, scaffolding, and drug release based on the side-by-side morphology and surface chemistry of the two sides [95].

#### 4.2.5. Multi-Jet Electrospinning

Multi-jet electrospinning is utilized to delineate a system in which the solution is fed into and then ejected directly from a number of respective capillaries or nozzles. Every nozzle, such as a needle, tip, hole, or channel, creates a single Taylor cone or a bubble in a certain situation. The electrospinning process could be more efficient by negatively charging the solution or positively charging the collector. Despite some downsides, multiple-nozzle electrospinning is still frequently employed for commercial NF fabrication due to the setup’s simplicity, versatility, and enhanced control over the distribution of the fibers [96,97]. Mohammadi et al. constructed 3D NF hybrid scaffolds made from PCL, PVA, and CS by employing a multi-jet electrospinning technique to pursue a biomimetic approach [98]. Table 2 summarizes the different types of electrospun fabrication techniques that have been recorded for bone regeneration.

## 5. Chitin-Based Electrospun Nanofibers in Bone Regeneration

Naturally, CT occurs as NFs; the characteristic linear structures of these NFs are due to their high degree of crystallinity and NF organization with the proteins that serve as a matrix [31]. CT NFs possesses significant biological activities such as anti-inflammatory, skin regenerative, and osteogenic characteristics and are applied in cosmetics, BTE, and other biomedical fields [106,113]. For instance, CT butyrate (CT-B)-coated nylon-6 NFs were fabricated by employing single-spinneret electrospinning with an amalgamation of CT-B and nylon-6 solution to form composite NFs; these NFs were evaluated for their cytocompatibility and bone-forming capability. Compared with the controls, the composite NFs enhanced cell proliferation and had a greater capacity to expedite the deposition of a calcium compound on the fiber surface. The results established that CT-B has a significant effect on the hydrophilicity, bone-forming ability, and cell biocompatibility of nylon-6 nanofibrous scaffolds [108]. CT NFs can be supported for improved mechanical properties with a stiff NF framework [114].

## 6. Chitosan-Based Electrospun Nanofibers as a Scaffold for Bone Tissue Regeneration

Glycosaminoglycans (GAGs) are essential for the bone microfield as, alongside Col and proteoglycans, they constitute one of the major organic components of the bone. CS is structurally analogous to GAGs and can be used as a substitute to imitate the organic phase of the bone ECM [115,116]. This ability of CS, combined with other bone ECM inorganic phase substitutes (bio-glass, HAp, etc.) in the form of ENFs, can lead to increased scaffold efficacy [117]. This combination also improves the scaffolds’ material and cellular regenerative capabilities for application in bone tissue rejuvenation, which are discussed in the following sections.

### 6.1. Chitosan-Based Electrospun Nanofibers: Material Characteristics

#### 6.1.1. Surface Topography

Bone has an uneven surface with pores and bumps and anisotropic surface morphology [118]. CS can provide a similar structure as it has the potential to alter the surface topography of a scaffold to favor the adhesion and proliferation of osteoblasts in BTE. Xu et al. prepared a PLA/CS core-shell ENF scaffold for BTE that had an outer shell of CS with island-like topographical features in the PLA/CS NFs that was made possible by controlling the temperature of the electrospinning process. SEM analysis of the NFs revealed that the diameter of the PLA NFs decreased with the addition of CS. The porosity of pristine PLA was 85 ± 5%, whereas that of a PLA/CS island-like mat was 93 ± 4%, implying easy cell communication and distribution [44]. The NFs’ distances and orientations are critical in acting as a guide for cell spreading. If the angle formed by two intersecting NFs is acute, it allows for greater capillary action of cells, establishing an NF scaffold as a stable, supportive material for tissue formation. Another team performed orientation analysis on electrospun polyamide-6/CS (PA6/CS) NFs, and the results revealed an alignment angle f of 0.68 ± 0.28, indicating that the NFs’ alignment degree was low. This could improve the migration and dissemination of regenerated cells [119,120].

#### 6.1.2. Mechanical Properties

Since bone is frequently subjected to significant mechanical stress, a suitable scaffold must be employed to cure a major bone defect completely. The intrinsic mechanical strength of CS alone as an NF scaffold produced by electrospinning is limited. To amplify its strength, either the parameters of the electrospinning process are altered [121] or the biopolymer is mixed with synthetic polymers or bioceramics to improve mechanical stability. Sedghi et al. grafted PCL to CS and obtained CS/PCL NFs through electrospinning. The mechanical strength of the NFs was analyzed using a stress–strain curve, and the authors obtained 20.1 ± 3.6 MPa, 17.1 ± 2.3 MPa, and 8.4 ± 2.2 MPa as tensile strength, Young’s modulus, and compressive strength, respectively [115]. Similarly, Liu et al. created PLLA/CS NFs by combining CS and PLLA. In dry conditions, the tensile strength and Young’s modulus of PLLA/CS nanofibers with a 0.075 mg/mL CS concentration were 3.17 ± 0.35 MPa and 1.24 ± 0.012 GPa, respectively. These values were 96.9% and 67.6% higher than for pristine PLLA [122].

#### 6.1.3. Wettability

Because of the presence of amino groups in the polymer, CS can easily absorb polar molecules such as water and is thus classified as hydrophilic [123]. When utilized as a scaffold for tissue engineering, a wettable polymer can adsorb the fluid in the ECM, hence sustaining cell adherence and proliferation. Adding CS to synthetic polymers that are typically hydrophobic improves wettability. For example, when mixed with PCL, CS increased the hydrophilicity of the ENF composite 4.6-fold in comparison with the PCL only NFs. Adding CS to PLA-co-PCL synthetic NFs increased the electrospun membrane’s water absorption capacity while slightly decreasing the water contact angle. This confirmed that CS improves the membrane’s hydrophilic behavior [124].

#### 6.1.4. Biodegradability

A scaffold must be gradually degraded until the completion of tissue regeneration. CS is biodegradable in nature, which is one of the properties that makes a scaffold biocompatible. The same property that makes it biocompatible is also a disadvantage because the destruction of CS by bodyily enzymes (lysozymes, chitinase, etc.) occurs at a relatively rapid rate [125]. Therefore, CS is mixed with synthetic polymers and bioceramics to slow the degradation. Tamburaci et al. conducted a lysozyme degradation test on their Si-doped nHAp reinforced bilayer CS nanocomposite membrane (CS/Si/nHAp) for 28 days [126]. The results showed that ENF CS scaffolds alone lost 79% of their initial weight, whereas the CS/Si/nHAp composite ENF scaffolds lost just 50% of their initial weight. CS elicits a synergistic approach to maintaining the degradability of a scaffold since CS is also used to improve the degradability of a synthetic polymer. In another study, PCL/CS/κ-C NF scaffolds were synthesized and in vitro degradation analysis was conducted at different timepoints by immersing them in two different solutions (Tris-HCL and PBS). The results demonstrated that addition of natural polysaccharides, such as CS, significantly increased the rate of degradation of the PCL/CS/κ-C NF scaffolds [127].

#### 6.1.5. Swelling Behavior

Swelling studies of a scaffold are conducted to ensure its stability in an aqueous environment. More than optimal swelling can lead to a loss of structural integrity, whereas a less than optimal swelling can hinder the transport of oxygen and nutrients within the scaffold [128]. The swelling ratio increases as the concentration of any hydrophilic group increases; CS, a hydrophilic chemical, is increasingly being employed to boost the swelling ability of scaffolds. When CS was fabricated into NFs, either alone or in combination with biopolymers such as curdlan, the swelling ability of the NFs was increased by between 300 and 350%. This swelling range indicated a higher surface area for improved cell adhesion and a larger pore size between NFs for the easy movement of nutrients and oxygen [129]. Salim et al. fabricated PVA-HA–CS–HAp ENF scaffolds and reported that addition of CS to the NF scaffolds enhanced the swelling ability by up to 240%, overcoming the HAp effect in swelling. This effect was predominantly due to the hydrophilic groups present in CS [130].

### 6.2. Impact of Chitosan-Based Electrospun Nanofibers on Biological Characterizations

Bone progenitor cells and osteoblasts can adhere to CS as it consists of the GAGs involved in various interactions with receptors, cell-adhesion molecules, and cytokines [131]. Composites utilizing CS can regulate wettability-sensitive integrins and increase the expression of OCN and osteoprotegerin [132]. In terms of proliferation, CS has been reported to hasten proliferation via upregulation of neural cell adhesion molecule (CD56) and tissue-type plasminogen activator. It has also been elucidated that CS can phosphorylate Smad 1/5/8, which is involved in a critical bone-remodeling regulatory pathway, i.e., the TGF-β signaling pathway. CS NFs also have the potential to activate the Runx2-mediated signaling pathway, upregulating ALP and OCN activity. This results in progenitor-cell differentiation and osteoblast maturation [133,134,135,136]. In this section, we elaborate on the recent studies involving CS NF composites in term of cell adhesion, proliferation, and differentiation.

#### 6.2.1. Cellular Adhesion

The proper establishment of cells onto and into scaffolds is necessary for tissue engineering. This can be ensured by using biomaterials to improve cell adhesion by increasing protein adsorption on their attachment sites [137]. Additionally, integrins are transmembrane receptors that aid in the process of cell attachment by enabling the exchange of signals between cells and the ECM [138]. Jing et al. fabricated Shish-kebab-structured CS/PCL NF scaffolds to provide integrin binding sites on the scaffolds, as the synthetic polymer PCL alone failed to offer the binding sites. This property and CS’s innate hydrophilicity enabled better cell adhesion of osteoblast-like MG63 cells [139]. Another team reported an increase in adhesion of MC3T3-E1 cells (mouse preosteoblastic cells) on PLLA NF scaffolds after the addition of CS into the electrospinning mixture. The increased adhesion was attributed to the improved hydrophilicity, which in turn could lead to increased protein adsorption into the NFs. The addition of CS also increased the surface area of the NFs, leading to the attachment of a significantly greater number of cells onto the exposed area [122].

#### 6.2.2. Cell Proliferation

After the proper attachment of cells, cell proliferation and spreading are crucial for tissue formation. To secure the proliferation of osteoblasts, the scaffolds need to be biocompatible, mimicking and sustaining a bone microenvironment favorable for the growth of stem cells and osteoblasts. CS derivatives have been reported to reduce intracellular reactive oxygen species, thereby promoting the proliferation and differentiation of mesenchymal stem cells (MSCs) [140]. Randomly aligned porous NFs are preferred for osteoprogenitor adhesion and proliferation. Random alignment warrants easy spreading and the pores enable the easy transport of nutrients and proteins into the scaffolds, facilitating the excretion of cellular wastes from the NF scaffolds [141,142]. Synthesized carboxymethyl CS (CMCS)/PCL ENF scaffolds were associated with a higher proliferation rate for osteoblast-like MG63 cells than other groups [143].

#### 6.2.3. Cell Differentiation

After adhesion and proliferation comes the differentiation of cells. MSCs are multi-progenitor cells that can be differentiated into the desired lineage of cells under conditions that stimulate the transition [144,145]. In the case of BTE, these progenitor cells are being used to treat bone defects by differentiating them into osteoblasts and affiliated cells that will aid in the regeneration process. The differentiation of these MSCs into osteoblasts can be achieved by regulating multiple signaling pathways, including the TGF-β, MAPK, Wnt, and BMP and notch pathways [146,147,148]. The activation of such pathways can be verified by checking the expression of various biomarkers, such as Runx2, osteocalcin (OCN), osteopontin (OPN), alkaline phosphatase (ALP), etc. The differentiation of cells can also be mediated by posttranscriptional regulators such as microRNAs [149,150]. CS ENFs offer an ideal microenvironment for the differentiation of attached MSCs into osteoprogenitor cells and osteoblasts. MC3T3-E1 cells that were grown on zein (Z) and CS integrated with polyurethane (PU) associated with functionalized multiwalled carbon nanotubes (Z/CS/PU/MCNT) ENFs showed an increased ALP activity and an increase in OPN and OCN expression. The results indicated that the CS biopolymer and carbon nanotubes promoted the osteoinductive potential of the scaffolds [151]. In another study, CS and platelet-rich fibrin (PFR) in CS/PCL/PFR ENFs induced a positive synergistic effect on the osteogenic differentiation of MSCs. Additionally, the presence of CS in the shell layer of the coaxially electrospun ENFs signified increased adhesion, which led to the proper proliferation and differentiation of the human MSCs [152]. Table 3 summarizes the various types of CS-based NF scaffolds that have been used in bone rejuvenation studies.

## 7. Chitosan-Based Electrospun Nanofibers as a Delivery Agent for Drugs and Small Biomolecules to Promote Bone Tissue Regeneration

Apart from serving as scaffolds, the CS ENF system can also be utilized to deliver drugs and small biomolecules that can promote osteoblastogenesis or decrease osteoclastogenesis. CS serves as a good drug delivery agent as its biodegradability enables the conditional release of drugs or biomolecules carried on/within the ENFs [159]. In the following sections we discuss recent research involving CS-based ENFs as a carrier in the delivery of drugs or small biomolecules.

### 7.1. Drug Delivery Using Chitosan-Based Electrospun Nanofibers

Antibiotics are often incorporated into nanocarriers for drug delivery to prevent infection at the defect site. Moreover, some antibiotics are reported to have osteogenic effects [160]. Delivery of such antibiotics via CS ENF carriers would have a synergistic effect on the development of bone tissue while simultaneously preventing infection. Topsakal et al., in their PU/CS/ β-TCP ENFs as a drug carrier, used amoxicillin (AMX) as the drug of choice. The team reported an encapsulating drug efficiency of 64% and a drug loading efficiency of 61.96%, with a controlled release of AMX. On comparative analysis with an analogous study, the drug release percentage was found to be 68% over 21 days [161]. Risedronate is a nitrogen-containing bisphosphonate that inhibits osteoclast formation by disrupting the RANKL pathway [162]. Since the CS nanosized fibers took advantage of the enhanced surface area brought about by electrospinning, they improved the drug’s solubility in the human system. To retard the burst drug release percentage, bioceramics such as bioactive glass (BG) can be added. El-Okaily et al. utilized CS/PVA ENFs as a drug carrier, to which BG was added to ensure the sustained and prolonged release of risedronate [163]. Simvastatin stimulates osteogenesis, predominantly through the Ras-PI3K-Akt/MAPK signaling pathway, which boosts BMP-2 expression. BMP-2 promoted the expression of a series of genes that are responsible for osteoblast differentiation and bone formation through Runx2 [164,165]. Ghadri et al. reported on the local release of simvastatin by utilizing CS ENFs in vivo, demonstrating bone growth in a rat calvarial defect [166].

### 7.2. Growth Factor Delivery Using Chitosan-Based Electrospun Nanofibers

Small biomolecules such as nucleic acids, growth factors, and hormones are being used to improve the innate tissue regeneration capability of the body. These small biomolecules are susceptible to degradation by bodily enzymes before they reach their site of action. In order to prevent this, nanocarriers are used, which can aid in keeping the biomolecules intact. Romero et al. reported on the simultaneous delivery of FGF-2 and TGF-β1, along with adipose-derived stem cells using heparin–CS-ENF-coated allografts. The growth factors were adsorbed onto the heparin-terminated polyelectrolyte multilayers on CS-ENF-coated allografts. The average amounts of FGF2 and TGF-β1 that were loaded onto the NF allografts from the 1000 ng/mL solutions were 127 ± 14 and 322 ± 32 ng, respectively [167].

## 8. Surface Functionalization of Chitosan-Based Nanofibers for BTE

Surface functionalization is the process of altering a surface’s existing physical, chemical, or biological properties. The modifications can enhance the scaffold’s cytocompatibility and biocompatibility, which could have the effect of enhancing the scaffold’s biointerface and trigger several cellular processes [154].

CS ENFs can be modified superficially using synthetic polymers and bioceramics, since CS has a disadvantage, i.e., high biodegradability, that could lead to a lack of mechanical strength. Surface modification of CS ENFs can help reconcile between good biodegradability and poor mechanical strength. Surface alteration can be achieved using various methods, namely crosslinking (chemical and physical modification), plasma treatment, the wet chemical method, graft polymerization, and layer-by-layer modification. The following sections discuss the various surface modification methods involved after synthesizing CS ENFs [168,169].

### 8.1. Crosslinking (Chemical and Physical Modifications)

The crosslinking of NFs can improve the thermal and mechanical properties of the scaffolds; crosslinking can also be applied to adjust the hydrophilic properties of the ENFs. There are two main categories of crosslinking: chemical and physical. Chemical crosslinking can develop due to various reactions similar to the Maillard and Schiff base reactions, i.e., a double proton transfer reaction, condensation reaction, hydrolysis, neutralization, etc., [154,168,169]. Mahdian-Dehkordi et al. used borax as a crosslinking agent on CS/PVA ENFs and reported increased tensile strength and hydrolysis resistance [170]. These effects induced by chemical crosslinking can aid in increasing the life of the scaffolds, which could lead to a consequent increase in the tissue-specific cell population over time. On the other hand, physical crosslinking represents reversible interactions formed via non-covalent bond formations such as electrostatic interactions, Van der Waals forces, and pi interactions [171,172]. Habibi et al. evaluated the ability of montmorillonite (MMT) as a crosslinker in their CS/Gel ENFs. From the FTIR analysis, the team inferred that the NFs were physically crosslinked with interactions between both CS and Gel counterparts. MMT was also reported to improve the electro spinnability of the NFs [173].

### 8.2. Plasma Treatment

Plasma treatment allows modification of the surface without affecting a scaffold’s bulk properties [174]. It can also be used to improve the surface area of the scaffolds at a nano/microscale without applying any hazardous chemicals. Plasma treatment of coral-nanoparticles (CL-NPs) incorporated into CS/polyethylene oxide (PEO) ENFs utilized the dielectric barrier discharge principle to induce plasma treatment for surface functionalizing the NF scaffolds with argon, nitrogen, and dry air as plasma ingredients. Nitrogen- and air-treated plasmas were reported to increase the NFs to 1.8 MPa, whereas the tensile strength of argon-treated NFs was 1.3 MPa. The surface of the ENFs was functionalized by incorporating oxygen- and nitrogen-containing groups. Nitrogen and air plasmas made the etching effect possible, increasing the friction between the CL nanoparticles and PEO/CS NFs and improving cell adhesion [175]. As a unit, these NFs were described to improve osteointegration, osteoinduction, and osteoconduction.

### 8.3. Wet Chemical Method

**Figure 5:**
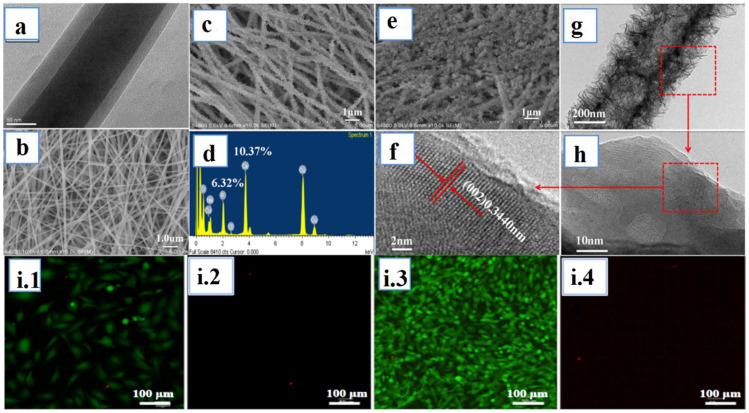
(**a**) TEM image of CS/PEO-Gel NFs, (**b**) SEM image of crosslinked CS/PEO-Gel NFs, (**c**) SEM image of HAp/CS/PEO-Gel NFs at cycle C1, (**d**) the EDS spectrum of the HAp/CS/PEO-Gel NFs, (**e**) SEM image of HAp/CS/PEO- Gel NFs at cycle C2, (**f**) HRTEM image of HAp/CS/PEO-Gel NFs, (**g**) TEM image of HAp/CS/PEO-Gel NFs, (**h**) HRTEM image of HAp/CS/PEO-Gel NFs, (**i**) fluorescence images of live (green) and dead (red) MG63 cells cultured on 48-well plates with the leach liquor of HAp/CS/PEO-Gel NFs (C1) at 24 h (**i.1**,**i.2**) and 48 h (**i.3**,**i.4**). ^©^Copyright 2018, Elsevier publishing group [178].

Wet chemical treatments can modify the structure of NFs as the material is completely submerged in an acidic or basic solution that adds hydroxyl and carboxylic acid groups onto the fiber material. Based on the immersion period, the material properties can be modified and tweaked to the desired extent [176]. Other merits include the minimal time required for surface modification in relation to other conventional precipitation methods; this method also offers a stable conjugation between HA and the CS NFs, along with promoting ossification [177]. To replicate both the chemical composition and the milieu of innate bone, Chen et al. constructed HAp/CS-PEO/Gel core-shell ENF composite scaffolds. Firstly, a CS-PEO/Gel NF mat was created using the coaxial electrospinning approach, which produced a 3D porous architecture that improved the environment for cell growth. Secondly, to further prompt osteoblast cell proliferation, HAp was deposited onto the surface of CS-PEO/Gel NFs via a wet chemical process. Compared with CS-PEO NFs, Gel NFs, and CS-PEO-Gel composite NFs, the mineralization efficacy of CS-PEO/Gel core-shell structured NFs was elevated. This was possible because CS served as the shell and the abundant functional amino groups on the CS molecules improved the mineralization ability. Notably, HAp/CS-PEO/Gel NFs substantially improved the viability of the MG63 cell lines used in culture (Figure 5) [178]. Thus, the wet chemical method is a convenient surface modification method.

### 8.4. Graft Polymerization

Graft polymers can also be referred to as graft copolymers due to the presence of at least two different types of monomer units, such as grafted side chains that differ structurally from the main chain, i.e., CS polymer [179]. Graft polymerization can aid in improving surface functionality while maintaining the integrity of the CS ENFs scaffolds. The β-cyclodextrin-grafted CS ENFs (β-CD/CS-ENFs) synthesized by Lee et al. exploited the surface grafting method. Their report indicated that the graft polymerization method increased the scaffold’s thermal stability and hydrophobicity and allowed a sustained release profile [180]. Thus, this method of combining hydrophilic (CS) and hydrophobic components through surface grafting to obtain the ideal adsorption of nutrients and optimal degradation as a drug carrier for hydrophobic drugs might have potential for use in BTE.

### 8.5. Layer-by-Layer Self-Assembly

Layer-by-layer (LBL) self-assembly allows multilayer deposition of inorganic/organic polymers onto the ENFs surface. The ENFs act as the substrate upon which the subsequent layering is obtained via electrostatic self-assembly [181]. The LBL method of surface modification is used to improve the mechanistic properties of scaffolds, which leads to sustained or prolonged release of any osteogenic drug or small molecule in the field of BTE. Cheng et al. assessed the release kinetics of connective tissue growth factor (CTGF) and BMP-2 through SF/PCL/PVA core-shell NFs fabricated using coaxial electrospinning that were layered with CS in increasing amounts (20, 30, and 40 layers). They dipped SF/PCL/PVA NFs in positively charged CS, followed by dipping them in negatively charged CTGF, which resulted in LBL self-assembly. Experiments carried out in vitro and in vivo showed that the dual-drug release technique enhanced bone tissue healing [182]. The LBL method of surface modification offers the prospect of simultaneous dual administration of osteogenic and angiogenic drugs/small molecules that could result in a synergistic response for healing bone defects. In chemical functionalization methods, the degradation of scaffolds functionalized by chemicals can release toxic allergens. Therefore, physical functionalization methods are preferred to avoid the induction of any harsh reactions in physiological conditions [183].

## 9. Role of Derivatives of Chitin- and Chitosan-Based Nanofibers in Bone Tissue Engineering

The main component of CT is produced from crustaceans, such as crabs, shrimp, and others. Pristine CT polymer is a firmly complex structure, making it difficult to dissolve in common solvents [184]. CT is thus dissolved in organic solvents that might be toxic in nature. Even if CT dissolves completely, as an electrospinning solution it is highly viscous in nature, which results in NFs with intermittent beads formed in between them. Certain derivatives of CT offer properties such as low viscosity, which ensures ease of electrospinning. In contrast, other derivatives can be applied with other polymers to improve the mechanical strength of scaffolds, exploiting the bioactive nature of the derivatives of CT [185,186].

Propionylation is among the most recent methods for obtaining a derivative of CT. Propionylation triggers a change in the structure of CT by converting its hydroxy groups into acyloxy groups. By adding CT powder to propionic anhydride in the presence of a catalyst, amorphization of the crystalline CT occurs [186]. CT propionate (CT-P) has better solubility in a combination of mild solvents such as ethanol and water, leading to a less viscous solution than CT in organic solvents. Zhong et al. utilized these merits in their CT-P/PEO NF mats and reported an increase in Young’s modulus and maximum strength, which were 124.8 ± 21.7 MPa and 12.2 ± 1.9 MPa, respectively, in a specific ratio in comparison with pure PEO. Results from a surface wettability test showed that the NF mats’ hydrophobic qualities were increased compared with CT alone (CT is highly hydrophilic) and that their thermal properties were also increased [187,188]. CT-B is another derivative; it is obtained by mixing CT in a solution of butyric acid and trifluoroacetic anhydride. Improved CT solubility was achieved using butyric-acid-mediated O-acylation. Pant et al. fabricated CT-B-coated nylon-6 composite NFs utilizing a single-spinneret electrospinning approach. The findings of this study showed that the addition of CT-B had a significant impact on the hydrophilicity, capacity for bone growth, and cell biocompatibility of nylon-6 NFs [188].

**Figure 6:**
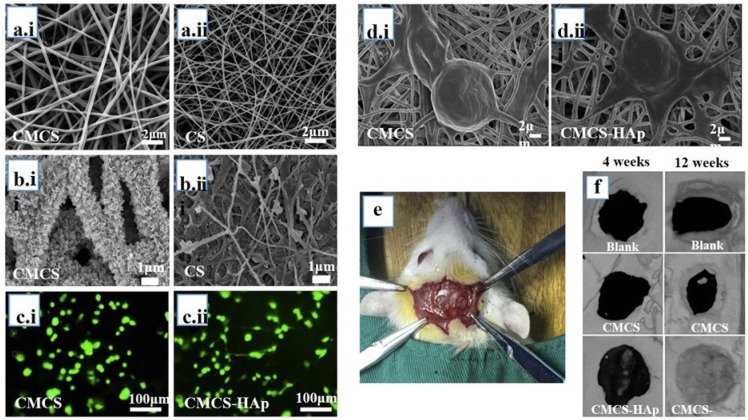
(**a**) Scanning electron micrographs (SEM) of glutaraldehyde-crosslinked CMCS (**a.i**) and CS (**a.ii**) NFs before SBF treatment, (**b**) SEM of glutaraldehyde-crosslinked CMCS (**b.i**) and CS (**b.ii**) NFs after SBF treatment, (**c**) LIVE/DEAD staining of mBMSCs cultivated for 12 h on CMCS (**c.i**) and CMCS-HAp (**c.ii**), (**d**) SEM images of cells cultured for 6 h on CMCS (**d.i**) and CMCS-HAp (**d.ii**), (**e**) a rat model of a calvarial bone defect, (**f**) micro-CT investigation of rat calvarial defect repair. ^©^Copyright 2018, Elsevier publishing group [189].

CS dissolved in any organic acid would result in a viscous solution; moreover, the obtained ENFs might have cytotoxic effects due to these solvents. Therefore, different derivatives of CS have also been experimented with in the past decade. ECM imitating functional groups, Ca^2+^ accumulation, and biomineralization can be increased according to the different derivations of CS. CMCS can be obtained by carboxymethylation of the hydroxyl and amine groups of CS; this molecule has increased solubility in water and is convenient for the processing of ENFs. With the help of PEO, Zhao et al. effectively created homogenous CMCS NFs. They achieved the ideal circumstances using PEO with a molecular weight of 1000 kDa and a voltage of 25 kV. Subsequently, they created HAp-coated CMCS ENFs through biomimetic mineralization using five times SBF immersion. According to the cell experiment, Runx2 and ALP gene expression levels on CMCS/HAp NFs were about 1.6- and 4.3-fold higher at 7 days and 5.1- and 10-fold higher at 14 days compared with CMCS alone samples. A critical-size rat calvarial bone defect model was used to study in vivo new bone formation via NF scaffolds. After a 12-week period of CMCS-HAp filling the defect, micro-CT and histological staining data revealed that the whole defect had been covered by new bone (Figure 6) [189]. In another study, Sharifi et al. synthesized CMCS by dissolving purified CS in isopropanol and sodium hydroxide followed by drop-wise addition of isopropanol and monochloroacetic acid mixture. The team reported that the PCL/CMCS solution prepared for electrospinning was less viscous than the PCL/CS electrospinning solution. Increased hydrophilic behavior was observed in the case of the CMCS-based NFs, as they have excess carboxylic acid groups in their backbone. The PCL/CMCS−10% scaffolds showed an increased affinity towards MG63 cells as CMCS presented with cell recognition sites; the NFs was also reported to increase the proliferation of the cells [190]. Similarly, Kasraei et al. created CMCS/PEO and CMCS/PVA ENFs and compared the nanostructures’ rheological and other properties. The increased amount of CMCS in the electrospinning solution of both scaffolds resulted in a decrease in the fiber diameter, as CMCS posed as an electrolyte, improving the electrical conductivity of the electrospinning solution. Beadless NFs were synthesized in both cases [191]. By combining CMCS with hydrophilic and hydrophobic polymers, biodegradability can be optimized for BTE applications.

## 10. Conclusions

BTE is highlighted as a capable alternative to conventional transplant techniques. Amongst the manufacturing processes available for constructing bone scaffolds, the electrospinning technique is quite simple and is employed chiefly to manufacture NFs with high surface areas and porosities. Incorporating CT or CS with other polymers and/or bioactive molecules produced osteostimulatory materials in the ENF scaffolds’ design, with reference to their mechanical strength, surface topography, cell attachment, proliferation, and osteoblast differentiation. Consequently, it can be inferred from thorough reviews of the literature that CT- and CS-based ENFs have become immensely significant in the field of BTE and are expected to bring about a revolutionary change in regenerative medicine.

## 11. Future Perspectives

Electrospinning technology has much potential for producing complex structures such as bone, cartilage, and osteochondral tissue for tissue engineering. ENFs have been analyzed and explored as regenerative medicine scaffolds and show great promise in biomedicine and clinical treatment. Various kinds of ENFs are also being constructed into scaffolds for bone growth due to their decisive superiority in multiple properties, such as ease of functionalization, large surface areas, excellent mechanical properties, and easy availability. The orientation of ENFs can direct the differentiation status and morphology of attached cells. In general, ENF scaffolds can foster osteogenesis with the abovementioned elements.

Nevertheless, this technology is still nascent, and some facets require further investigation. Some critical research and technical problems must be addressed to improve the development of electrospinning scaffolds in BTE applications. For example, optimization of electrospinning addition and dispersion procedures, the influence of material load levels on mechanical and biological traits, utilization of existing electrospinning methods to create multifunctional scaffolds, the use of molecular biology techniques to investigate the detailed mechanism underlying the formation of bone formation, and the validation of the effectiveness and security for clinical translation are warranted.

## Figures and Tables

**Table 1:** Summary of the different scaffold fabrication techniques.

Fabrication Techniques	Working Principles	Examples of Parameter Ranges	Significances	Associated Limitations	References
Freeze drying	Scaffolds obtained by the removal of water and/or other solvents by sublimation in vacuum condition.	Scaffolds were freeze dried for 2 h at a temperature of −20 °C, attained at a rate of −0.5 °C/min, followed by sublimation in vacuum under 80 mTorr pressure at 0 °C.	Enabled obtainment of highly porous scaffolds i.e., allowed facile cell migration. Increased surface volume ratio.	Highly energy consuming process Might require use of toxic solvents that could lead to release of toxic fumes.	[55,56]
Phase separation	Phase separation can either be induced by the addition of a non-solvent or a solvent with respect to the polymer utilized, or can be induced by mass transfer and/or heat transfer.	Thermally induced phase separation required freezing scaffolds at very low temperatures (−20 °C), followed by lyophilization.	Adjustable pore morphology. Supported higher cell proliferation and cell density.	When used as a synthesis method for fabricating nanofibers, it resulted in non-uniform fibers. Complex process.	[57,58]
Gas foaming	Materials are placed in a gas chamber and subsequent increase in pressure resulted in the imbibement of the gas into the material. Lowering the pressure back to atmospheric pressure resulted in pores.	During the imbibing phase, the pressure must be as high as 800 psi. Wax/sugar/salts can be used to sustain the connectivity of the pores.	Organic solvents and high temperatures were not required.	Produced scaffold structures that might collapse on exposure to culture media for a longer duration.	[43,59]
Decellularized matrix	Decellularized scaffolds were obtained by removal of cells to the most extent by enzyme, chemical digestion, or physical removal, leaving behind an integral ECM. All three method of removals could also be utilized in preparing the scaffolds.	For physical removal Freeze-thawing temperatures: −80 °C to 37 °C Solution used to freeze-thaw: Tris-buffer Number of freeze-thaw cycles: 5 For Enzyme digestion Trypsin EDTA wash followed by enzyme digestion using DNases and RNases. Final PBS wash	Virtual bone ECM mimicry	Scale-up is not feasible. Chemical decellularizing agents such as surfactants including SDS are cytotoxic and hence if not thoroughly washed can result in cytotoxicity.	[60,61]
Selective Laser Sintering (SLS)	It is a 3D-printing process based on additive manufacturing of a computer aided design (CAD). It involves sintering of micron-sized materials in its raw form using a controlled LASER, followed by its deposition layer-over-layer over a bed of a non-reactive material.	CAD model as an .STL file with required dimensions fed into the SLS apparatus. LASER power: 2–3 W Hatch distance: 60 µm Scanning speed: 80 mm/s	3D scaffolds of desired shape and size could be fabricated. Pore size of the scaffolds can be made homogenous.	Expensive process. Time consuming. Requires high temperature conditions. High maintenance.	[62,63]
Fused Deposition Modelling (FDM)	Thermoplastic materials are fabricated into a 3D model by extruding it in the semi-molten state, with the desired dimensions onto a substrate where it solidifies.	CAD model as an .STL file with required dimensions fed into the FDM apparatus. Melting temperature: 210 °C Nozzle diameter: 400 µm Printing speed: 6000 mm/min Print bed temperature: 40 °C	Relatively simple and faster printing compared to SLS/SLA. Reduced carbon footprint. Low material cost.	Requires high temperature conditions. Less accurate in comparison with SLS.	[64,65,66]
Stereolithography based additive manufacturing	A mixture of photopolymer-resin along with a photo-initiator is layered over a substrate bed, and is exposed to a scrutinized light source, solidifying the area of interest, producing a 3D scaffold of desired dimensions.	CAD model as an .STL file with required dimensions fed into the SLA apparatus. Polymer resin and photo-initiator mixture are to be prepared without bubbles. If bubbles are present then it must be degassed.	Highly precise. The only photocuring technique.	Printing rate is relatively slow to other additive manufacturing methods. The cationic-polymer resins applicable for the process are limited. Low resolution.	[67,68,69,70]
Solvent casting and Particulate leaching	In Solvent Casting and Particulate leaching, porogen is added to the mould containing the polymer in its solvent. Once the solvent is evaporated, the porogen along with the polymer is then immersed in a solvent to remove the porogen.	Polymer solution: PCL in chloroform with BG nanoparticles dispersed in it. Porogen: Nacl (salt) particles. Temperature: room temperature. Evaporation time: 48 h Solvent to dissolve porogen: Immersion in deionized water for 5 days.	Cost effective Can produce highly porous scaffolds.	Can result in the formation of an outer denser layer and an inaccessible porous inner mass. Non-uniform dispersion of pores owing to the settling nature of the porogen.	[71,72,73,74]
Bioprinting	A bioprinter jet can work on various properties including thermal, piezo-electricity, pressure. The material is generally called as bioink and is deposited LbL over a substrate.	Stabilizing cartridge temperature: 25 °C Nozzle diameter: 400 µm Tip velocity: 5 mm/s Printing speed: 5–1800 mm/s Material viscosity: 1–6 × 10^7^ mPa/s Extrusion pressure: 120–180 kPa	Inkjet and micro-extrusion bioprinting are cost effective. Open nozzle LASER structure causes minimal damage to cells during the bioprinting process. Biomimicry can be achieved	LASER assisted bioprinting is a very expensive process. Time of fabrication is medium to very long.	[75,76,77]

**Table 2:** Summary of the different types of electrospinning techniques.

Types of Electrospinning Techniques/Schematic Representation	Surface Topography	Significances	Limitations	References
**SINGLE JET ELECTROSPINNING** 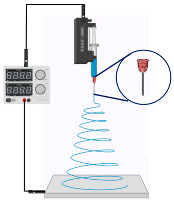	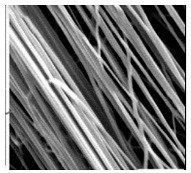	Large scale production Simplicity of process and functionalisation Ease of production and mechanical attributes of NFs produced Effective encapsulation of drugs/biomolecules	Clogging of needle Low scalability Time consuming Usage of volatile solvent might be hazardous to health	[79,99,100,101,102]
**CO-AXIAL** **ELECTROSPINNING** 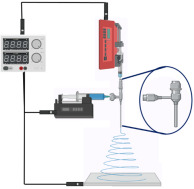	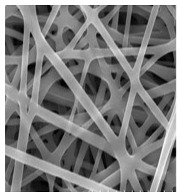	Can modify wettable property of NFs Combining attributes of different materials Constructing desirable structures	Developing coaxial electrospinning machinery for industrial application is challenging. Require many experimental trials with different parameter, making the process complicated.	[103,104,105,106]
**MULTI-JET ELECTROSPINNING** 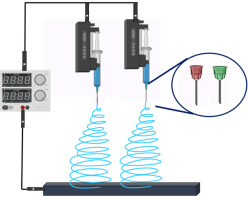	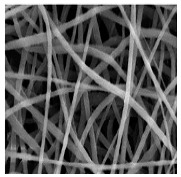	Augmented productivity Can be utilized for industrial applications.	Difficulty in regulation process Lower fiber quality owing to uneven fiber formation.	[97,107]
**BUBBLE ELECTROSPINNING** 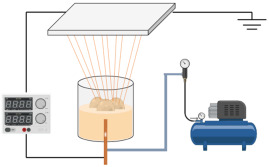	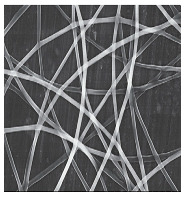	Can be utilized for large scale manufacturing. Needless technique hence prevents clogging. Can produce uniform fibers ranging from nanometres to micrometres.	High voltage supply Hefty price Swift solvent evaporation	[108,109]
**WIRE ELECTRODE ELECTROSPINNING** 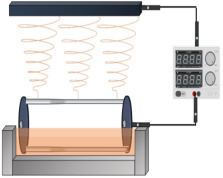	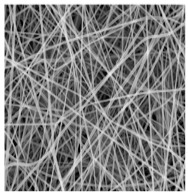	High productivity Employed for mass production. Can produce thin NFs of range 80–700 nm.	High voltage supply Hefty price	[110,111,112]

**Table 3:** Summary of the diverse CS-based nanofibrous scaffolds in BTE applications.

Chitosan Composites	Fabrication Methodologies	Fiber Alignment	Electrospinning Parameters	Cell Type	Results and Significance	Reference
PCL/Gel/CS-nHAp NFs scaffolds	Electrospinning of PCL: Gel: CS solution in the ratio of 80:10:10, followed by dipping and stirring in nHAp for surface functionalization	Random orientation	Syringe volume: 3 mL Needle tip diameter: 0.56 mm Voltage applied: 22 kV Flow rate: 0.1 mL/h Distance between needle and collector: 10 cm	Human osteoblastic cells and mouse fibroblastic cells.	In vitro data of MTT assay and DNA quantification revealed significant increase in cell proliferation. Roughness of the scaffolds improved adhesion of the polygonal shaped osteoblasts with well spread morphology. Osteoconductivity and biocompatibility were observed.	[153]
HA-coated CS/PEO NFs mats	Electrospinning of CS: PEO in the ratio of 4:1, followed by simultaneous neutralization and coating of the mats with HA	Random orientation	Syringe volume: 10 mL Flow rate: 1 mL/h Voltage applied: 20 kV Distance between needle and collector: 14 cm	Human normal dermal fibroblasts	There were increased cell adhesion and proliferation. SEM images revealed filopodia and cell extensions indicating cell-scaffold integration.	[154]
CS/PEO NFs membranes	Electrospinning of CS: PEO in the ratio of 95:5	Both random and aligned orientations of NFs	Syringe volume: 1 mL Needle gauge diameter: 4.699 mm Distance between needle and collector: 16 cm Voltage applied: 17.8 kV to 22 kV	Human osteoblastic cells and human embryonic mesenchymal progenitor cells.	There was a superlative degree of cell infiltration. This was confirmed by histological cryosectioning. The ultimate tensile strength of aligned NFs was found to be 13.58 ± 3.68 MPa whereas it was 7.5 ± 3.84 MPa for randomly oriented NFs.	[155]
PCL/CMCS NFs scaffolds	Electrospinning of CMCS: PCL in 3 subsequent ratios (5%, 10%, 15%), followed by BMP-2 immobilization via cold atmospheric plasma treatment	Random orientation	Syringe volume: 5 mL Distance between needle and collector: 16–20 cm Voltage applied: +18 to 30 kV Flow rate: 0.1–0.7 mL	Human bone marrow derived MSCs	There was increased in ALP activity. Increased Runx2 and Sox9 suggested osteoblast differentiation. Alizarin red staining confirmed calcium deposition onto the scaffold.	[156]
CS membranes	Electrospinning of CS with a degree of deacetylation of 71%, followed by its treatment using triethylamine (TA)/acetone and di-tert-butyl dicarbonate (DTBD) to remove residual acid salts of CS’s solvent.	Random orientation	Syringe volume: 10 mL Needle gauge diameter: 3.81 cm Distance between needle and collector: 15 cm Voltage applied: 26 kV Flow rate: 15 µL/min	Human osteoblastic cells	Mechanical tear test of the membrane concluded that the treated membranes provide with a secure barrier for bone graft materials. Histology photomicrographs exhibited obscured integration of the old bone and new bone at the bone defective site, indicating the increased efficiency of the membrane as an aid for BTE applications.	[157]
NFs CS coating and mineralized bone allograft (MBA) on the surface of AZ31 Magnesium alloy	Electrospinning of CS solution onto the Mg alloy, followed by post neutralization step in weak alkaline aqueous environment	Random orientation	Syringe volume: 5 mL Distance between needle and collector: 15 cm Voltage applied: 25 kV Flow rate: 1 mL/h Temperature: Room temperature	Fibroblast cells	There were homogenous surface morphology with highly interconnected pores, decreased water contact angle, and improved corrosion resistance of the alloy. There were increased cell proliferation on the surface of the alloy due to CS/MBA coating.	[158]

## Data Availability

Not applicable.
